# Uniform intrahepatic portal ammonia distribution in cirrhosis

**DOI:** 10.1038/s41598-025-21758-0

**Published:** 2025-10-01

**Authors:** Carsten Meyer, Olga Ramig, Narine Mesropyan, Patrick Kupczyk, Johannes Chang, Michael Praktiknjo, Julian Luetkens, Daniel Kuetting, Christian Jansen, Tatjana Dell

**Affiliations:** 1 Department of Diagnostic and Interventional Radiology and Quantitative Imaging Lab Bonn (QILaB), University Hospital Bonn, Venusberg-Campus 1, 53127 Bonn, Germany; 2 Department of Internal Medicine I, Center for Cirrhosis and Portal Hypertension Bonn (CCB), University Hospital Bonn, Venusberg-Campus 1, 53127 Bonn, Germany; 3 Department of Medicine B, Muenster University Hospital, Albert-Schweitzer-Campus 1, 48149 Muenster, Germany

**Keywords:** Transjugular intrahepatic portosystemic shunt (TIPS), Hepatic encephalopathy, Portosystemic overshunting, Ammonia, Cirrhosis, Diseases, Gastroenterology, Medical research

## Abstract

Lower rates of hepatic encephalopathy (HE) following left-sided transjugular intrahepatic portosystemic shunt (TIPS) placement have been hypothesized to stem from a distinct ammonia distribution within the portal venous system. This prospective study investigated ammonia concentrations at five portal and splanchnic venous sites in 50 fasting cirrhotic patients (20 female [40%]; mean age: 60.4 years) prior to TIPS implantation for ascites (33/50, 66%) or variceal bleeding (17/50, 34%). While ammonia levels were significantly higher in the superior mesenteric vein (mean: 143 µg/dl) compared to the splenic vein (mean: 66 µg/dl; *p* < 0.001), mean ammonia concentrations in the right (104 µg/dl) and left (107 µg/dl) portal vein branches were found to be equivalent (*p* = 0.008 for equivalence). No systematic differences between right and left portal vein ammonia were observed. These findings suggest that in fasting cirrhotic patients, local ammonia levels in the main portal vein branches do not differ significantly. Therefore, other factors likely contribute to any observed differences in HE rates related to TIPS placement site, warranting further investigation into alternative mechanisms.

## Introduction

Transjugular intrahepatic portosystemic shunt (TIPS) is an established, minimally invasive intervention for managing complications of portal hypertension, such as variceal bleeding and refractory ascites^1,2^. Despite its efficacy, post-TIPS hepatic encephalopathy (HE) remains a significant and frequent complication, with reported incidences ranging between 15% and 67%, impacting patients’ quality of life and prognosis^1,3–6^. Consequently, strategies to predict and prevent post-TIPS HE are a research priority. While medical prophylaxis and technical aspects like shunt diameter are explored^7–11^, the optimal portal vein puncture site for TIPS placement is also debated.

A meta-analysis by Zuo et al.^12^ suggested that TIPS implantation into the left portal vein (LPV) may be associated with lower rates of post-interventional HE compared to implantation in the right portal vein (RPV). The authors hypothesized this observation could result from a non-homogeneous distribution of ammonia, with higher concentrations favoring the right portal vein^12^. As hyperammonemia is a recognized neurotoxin and a key contributor to HE pathogenesis^13–15^, this hypothesis provides a plausible, yet unverified, mechanism for the observed differences in HE rates.

Therefore, this prospective study aimed to directly assess blood ammonia concentrations at multiple distinct sites within the portal and splanchnic venous systems of cirrhotic patients prior to TIPS creation, to investigate the validity of this proposed uneven ammonia distribution.

## Results

**Fig. 1:**
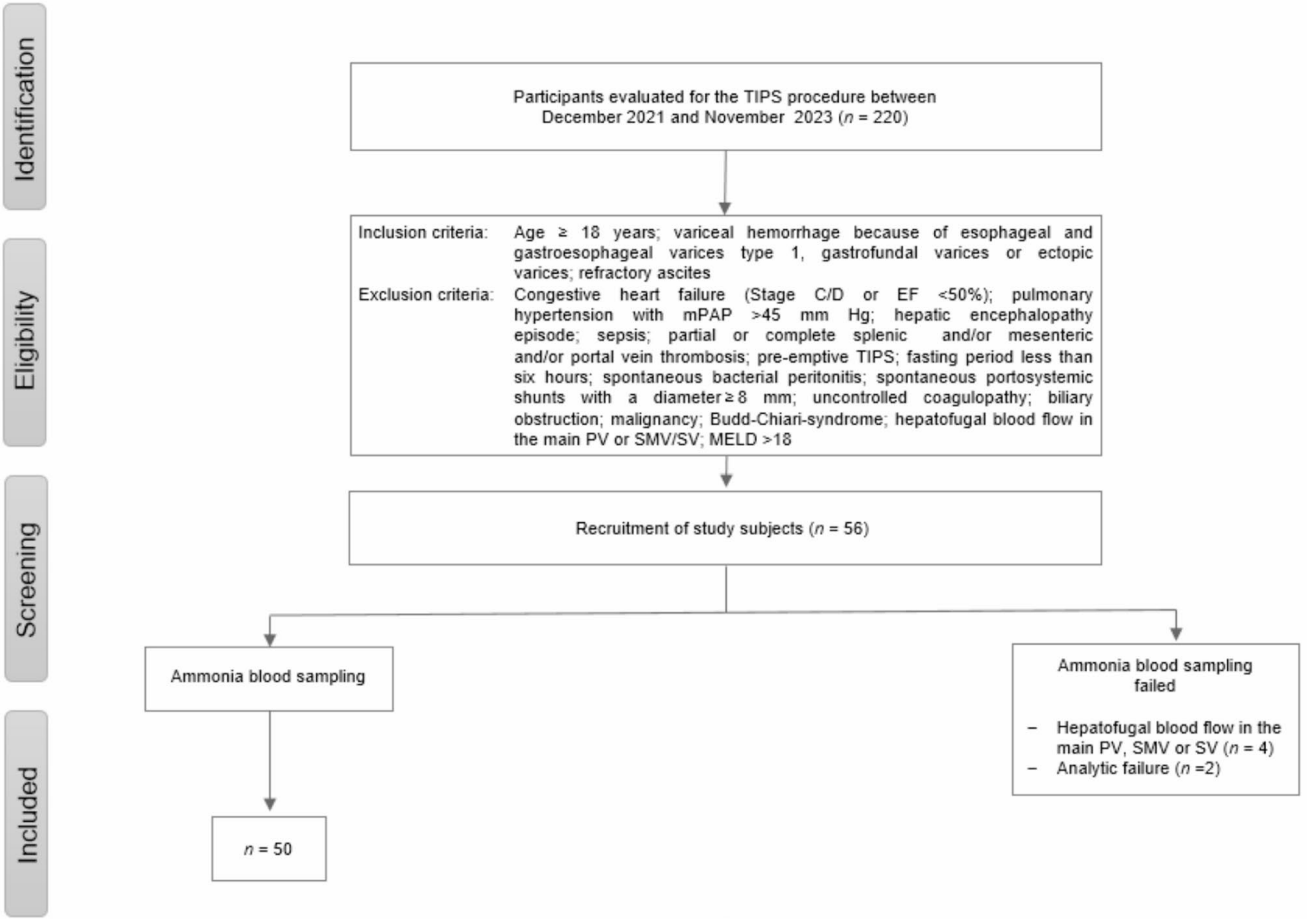
Flow diagram inclusion criteria (PV: Portal vein; SMV: Superior mesenteric vein; SV: Splenic vein).

A total of 50 consecutive patients with decompensated liver cirrhosis (20 female; mean age: 60.4 years, SD 12.5) undergoing TIPS were included in this study (Fig.1). The primary indications for TIPS were refractory or recurrent ascites in 33 patients (66%) and recurrent variceal bleeding in 17 patients (34%). Baseline patient characteristics are detailed in Table 1. In 19 patients (38%), portosystemic collateral veins were embolized prior to blood sampling.

**Table 1:** Characteristics in 50 Subjects.

Patient characteristics (*n* = 50)	
Sex*	
M	30 (60)
F	20 (40)
Mean age^†^ (60.4, SD 12.5)	
M	59.5 (11.5)
F	61.9 (14.0)
TIPS Indication*	
Ascites	33 (66.7)
Variceal bleeding	17 (33.3)
Variceal embolisation* (*n* = 19; 38)	
Gastric varices	8 (16)
(Para)Esophageal varices	5 (10)
Perisplenic Varices	5 (10)
Retroperitoneal Varices	1 (2)
Etiology of cirrhosis*	
Chronic alcohol abuse	26 (52)
Chronic viral hepatitis	3 (6)
Nonalcoholic fatty liver disease (NAFLD)	6 (12)
Autoimmune hepatitis	2 (4)
Drug toxicity	5 (10)
Cryptogenic	8 (16)
PSG^†^	
Pre-TIPS	20.4 (5.1)
Post-TIPS	8.1 (4.0)
Child-Pugh Class*	
A	10 (20)
B	35 (70)
C	5 (10)
MELD Score^†^	12.7 (5.1)

* Data are numbers of patients, with percentages in parentheses.

^†^ Data are mean with standard deviation in parentheses.

Abbreviations: TIPS, transjugular intrahepatic portosystemic shunt; NAFLD, nonalcoholic fatty liver disease; PSG, portosystemic pressure gradient; MELD, Model for End-Stage Liver Disease.

**Fig. 2:**
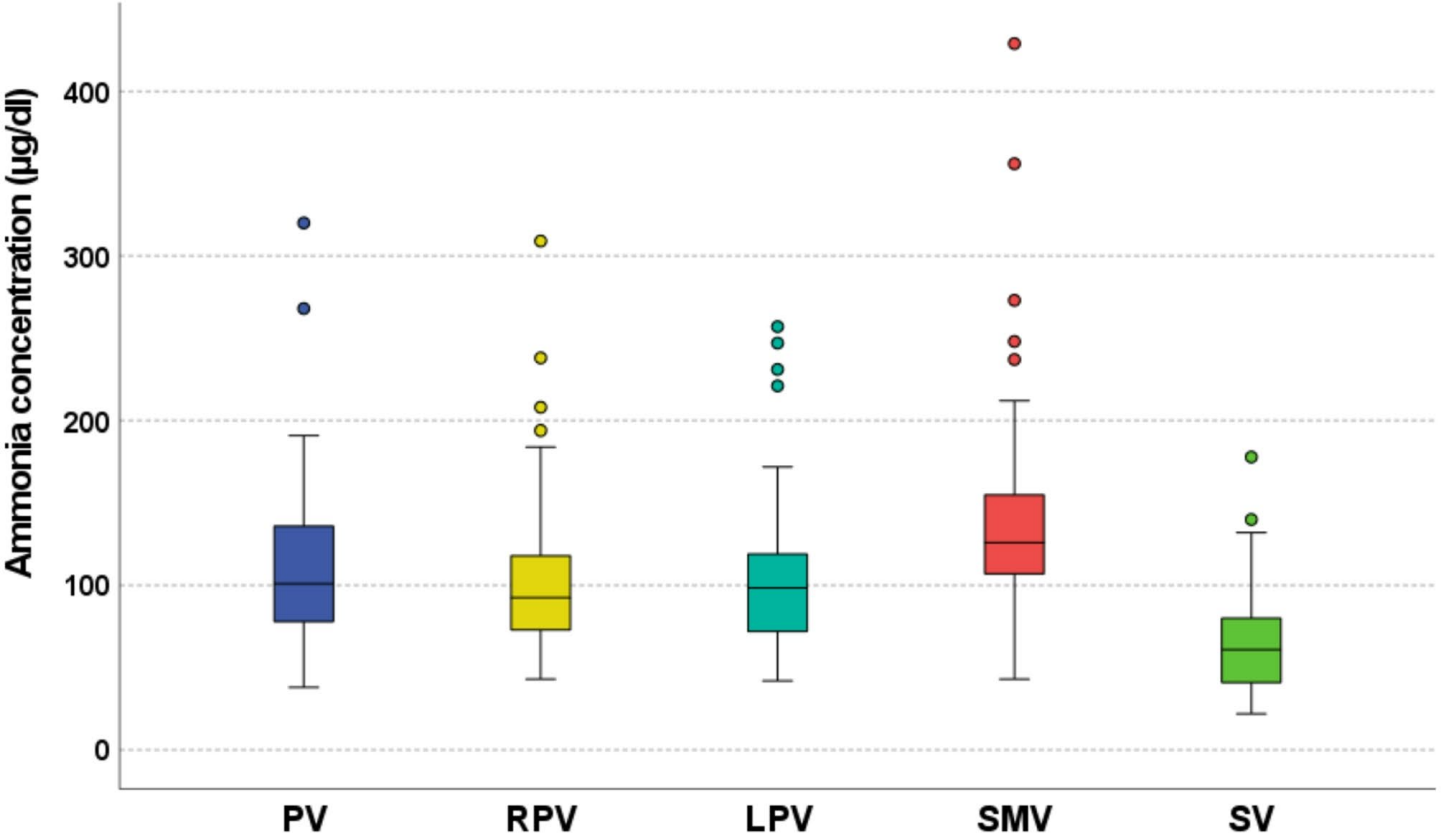
Distribution of ammonia concentration at the five sampling positions in the study population. Abbreviations: PV, main portal vein; RPV, right portal vein branch; LPV, left portal vein branch; SMV, superior mesenteric vein; SV, splenic vein.

The distribution of ammonia concentrations at the five distinct sampling sites is shown in Fig. 2. Ammonia levels were significantly higher in the superior mesenteric vein (SMV) (mean: 143 µg/dl, range: 43–429 µg/dl) compared to the splenic vein (SV) (mean: 66 µg/dl, range: 22–178 µg/dl; *p* < 0.001). The mean ammonia concentration in the main portal vein (PV) was 113 µg/dl (range: 38–320 µg/dl).

Regarding the portal vein branches, mean ammonia concentrations were 104 µg/dl (range: 43–309 µg/dl) in the right portal vein (RPV) and 107 µg/dl (range: 42–257 µg/dl) in the left portal vein (LPV). The Two One-Sided Tests (TOST) procedure confirmed statistical equivalence between RPV and LPV ammonia levels within the prospectively defined ± 9% margin (*p* = 0.008 for equivalence). The mean absolute difference between LPV and RPV ammonia concentrations was 3.0 µg/dl. Based on the overall mean ammonia level in the portal branches (105.5 µg/dl), this represents an average difference of approximately 2.8%.

**Fig. 3:**
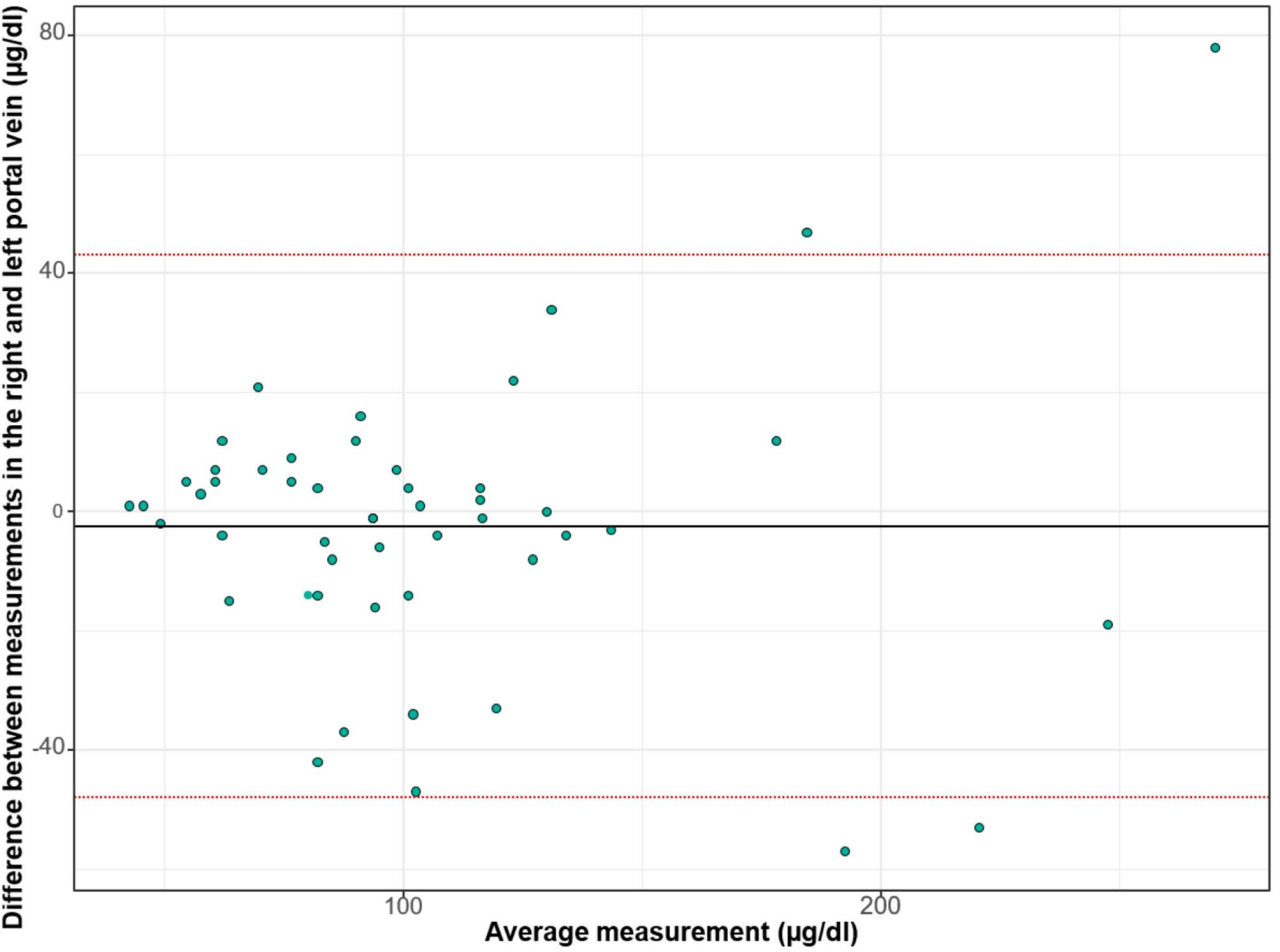
Bland–Altman plot comparing ammonia concentrations between the right and left portal vein branches. The mean bias was − 1.8 µg/dl, with 95% limits of agreement from − 47.7 to + 42.1 µg/dl.

**Fig. 4:**
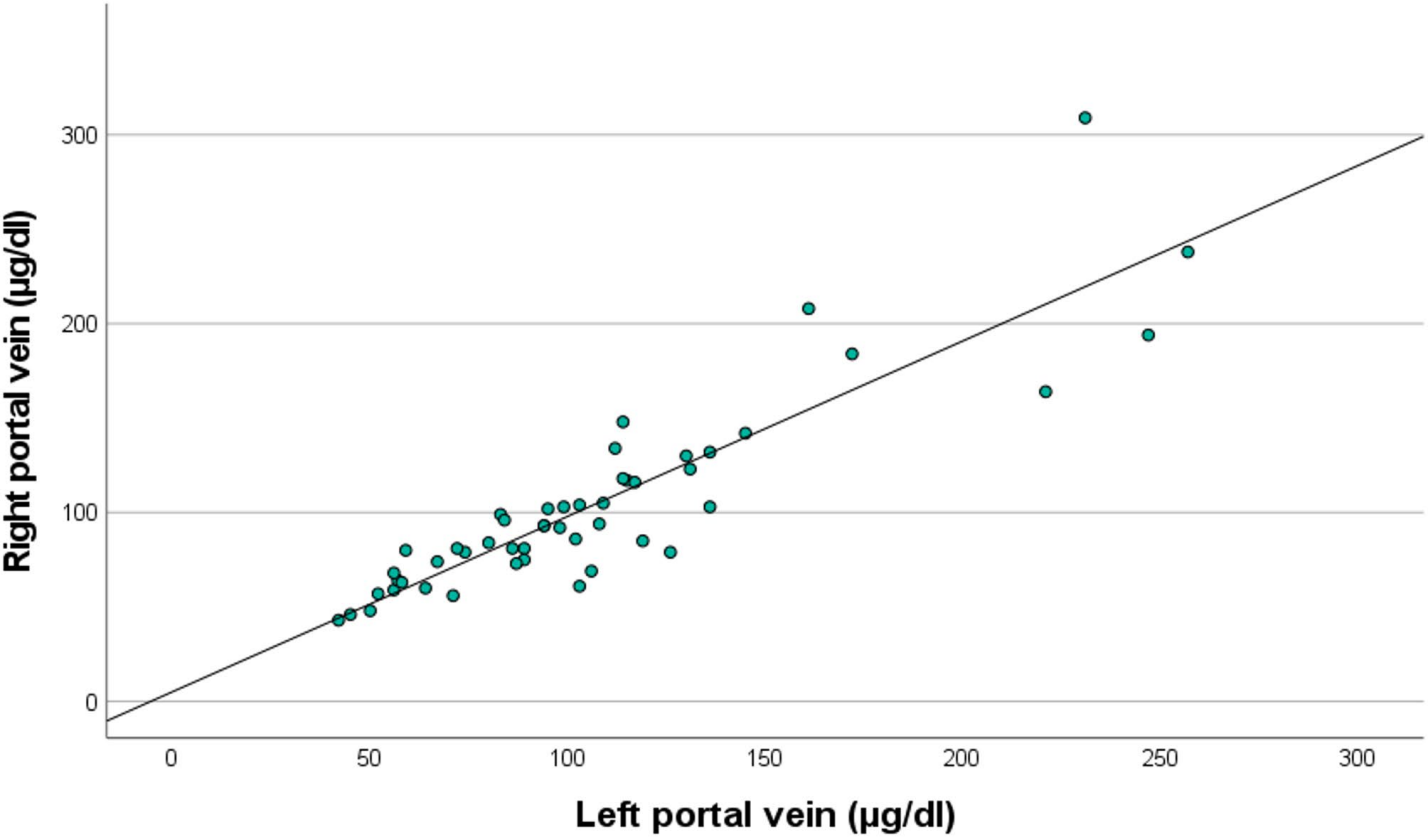
Scatter plot comparing ammonia concentrations in the right and left portal vein branches. Pearson correlation coefficient *r* = 0.801 (R² = 0.641, *p* < 0.001).

The relationship between RPV and LPV ammonia concentrations is further detailed in Fig. 3 (Bland-Altman plot) and Fig. 4 (scatter plot). The Bland–Altman analysis (Fig. 3) showed a mean bias of − 1.8 µg/dl, with 95% limits of agreement ranging from − 47.7 to + 42.1 µg/dl. No systematic differences were observed between the RPV and LPV measurements across the range of ammonia concentrations, although the scatter of individual differences tended to be wider at higher mean ammonia levels. Figure 4 illustrates the correlation between RPV and LPV ammonia concentrations, with a Pearson correlation coefficient of *r* = 0.801 (R² = 0.641, *p* < 0.001).

The mean baseline serum creatinine level was 1.43 mg/dl (median 1.03 mg/dl, range 0.43–7.87 mg/dl). Renal function was thus moderately impaired in some patients but overall within a spectrum representative of decompensated cirrhosis. There was no relevant correlation between serum creatinine and portal vein ammonia concentrations (*r* = 0.006).

## Discussion

Hepatic encephalopathy remains a significant challenge following TIPS placement, impacting patient outcomes and quality of life. While its incidence varies across studies, understanding and mitigating HE is a clinical priority. Ammonia, a gut-derived neurotoxin, plays a key role in the pathogenesis of HE by mechanisms including increased blood-brain barrier permeability and altered cerebral neurotransmission, particularly in the context of impaired hepatic clearance in cirrhosis^15^. The creation of a TIPS can exacerbate hyperammonemia by shunting unpurified portal blood directly into the systemic circulation, with the shunt volume potentially correlating with HE risk^16^.

The streamline flow in the portal system is a well known hypothesis by which blood from superior mesenteric vein flows preferentially to the right hepatic lobe, while splenic and inferior mesenteric veins divert preferentially to the left lobe^17^. The debate regarding optimal TIPS placement taking into account the aforementioned fact and its impact on post-TIPS HE is ongoing. A meta-analysis by Zuo et al. suggested lower HE rates with LPV-TIPS, hypothesizing this could be due to a “streamline” based non-homogeneous distribution of ammonia, with the LPV predominantly receiving lower-ammonia blood from the SV and the RPV higher-ammonia blood from the SMV^12^. This hypothesis provided a plausible, albeit unverified, rationale for investigating portal venous ammonia distribution. Our study was designed to directly test this premise by measuring ammonia at multiple distinct sites. While Zuo et al.‘s meta-analysis highlighted a potential difference^12^, it is important to note that its conclusions were based on a limited number of studies, primarily from a single geographical region, with some not readily accessible for independent review, and included animal data of questionable direct relevance to the clinical scenario^18^. In addition, the meta-analysis by Zuo et al. did not uniformly report stent calibers: in three of the included studies this information was not provided, and in another three studies two different stent diameters were used. As stent diameter is known to influence shunt hemodynamics and the risk of post-TIPS hepatic encephalopathy, this inconsistency further limits the interpretability of the pooled results. This underscored the need for direct physiological measurements in humans.

Previous studies on stent positioning and HE have yielded mixed results. While an earlier randomized trial using uncovered stents showed lower HE rates with left-sided TIPS^19^, and a propensity score analysis with 8 mm covered stents also suggested a benefit^20^, these findings are not universally replicated. Notably, a recent large retrospective analysis involving 193 patients found no significant difference in clinical outcomes, including HE rates, between left- and right-sided TIPS^21^. This further complicates the interpretation of the impact of shunt location alone.

Our findings demonstrate a significant difference in ammonia concentrations between the SMV and SV, consistent with the known physiology of ammonia production primarily in the gut supplied by the SMV. However, crucially, we found no statistically significant or clinically relevant difference in ammonia concentrations between the RPV and LPV in fasting cirrhotic patients. This contradicts the core assumption of the hypothesis proposed by Zuo et al. regarding differential ammonia shunting based on portal branch selection^12^. Nevertheless, our Bland–Altman analysis suggested that discrepancies between RPV and LPV ammonia concentrations may become more pronounced at higher absolute ammonia levels. Such a proportional bias could have clinical implications, given that elevated pre-TIPS ammonia is a recognized predictor of post-TIPS hepatic encephalopathy. Because only a small subset of our cohort exhibited markedly elevated ammonia levels, this trend cannot be considered statistically robust. We therefore highlight this as a hypothesis-generating finding that warrants confirmation in larger studies with stratification by baseline ammonia concentration.

Our results align more closely with the findings of Yang et al., who, through an analysis of portal venous flow distribution, observed that in the majority of patients (73%), blood from the SMV and SV was directed equally to both portal branches, suggesting a thorough mixing of portal inflow^22^. Although it should be noted that the assessment of portal blood distribution in this study has several limitations as it was based on a single portography and contrast media flow (primarily from splenic vein injection) may not perfectly reflect physiological blood flow dynamics, as contrast media can have different flow properties than blood due to density and viscosity differences^23,24^, the latter even changing at different temperatures and furthermore, details on injection protocols and their potential hemodynamic impact were not fully reported.

The absence of a significant ammonia gradient between the RPV and LPV in our cohort suggests that other factors may be more influential in determining post-TIPS HE rates. Liver volume relative to body weight has been associated with transplant-free survival after TIPS^25^, and the interplay between preserved liver volume, residual portal perfusion to non-shunted liver segments, and shunt characteristics (diameter, flow volume) might be more critical than the specific portal branch selected for puncture, especially if ammonia might be homogenously distributed by that point. The relative blood flow within the LPV and RPV, and thus the total amount of blood shunted, could indeed be a more pertinent factor, as suggested by some theories, though this was not directly quantified in relation to ammonia in our current study.

This study has several limitations. An important limitation of our statistical approach is the definition of the equivalence margin (± 9%) used for the TOST analysis. As no established clinically relevant threshold for ammonia concentration differences between portal vein branches exists in the literature, our margin was chosen pragmatically and derived from a power calculation to achieve sufficient statistical sensitivity with the available sample size. We acknowledge that this cutoff is arbitrary and should be interpreted with caution. Future studies with larger cohorts and clinical outcome correlation will be required to define a clinically meaningful minimal difference. Nonetheless, the choice of the TOST procedure remains crucial, as a non-significant paired t-test would merely indicate a lack of evidence for a difference, but cannot provide affirmative evidence for equivalence. The TOST design therefore allowed us to address the actual research hypothesis of “no relevant difference” between right and left portal vein ammonia concentrations. Secondly, all patients were fasting for at least six hours, which likely resulted in lower baseline SMV ammonia concentrations. Postprandial states, with increased intestinal ammonia production and potentially altered splanchnic hemodynamics^26,27^, could theoretically lead to different portal ammonia distribution patterns and this study did not assess postprandial changes. Thirdly, while we documented hepatopetal flow, we did not perform detailed quantification of relative blood flow volumes in the RPV and LPV pre-TIPS and correlated this with ammonia levels, which could have provided further insights. Finally, our study focused on pre-TIPS ammonia levels and did not correlate these with the actual incidence or severity of post-TIPS HE in our cohort, which would be a valuable next step. In addition, we acknowledge that we did not include detailed hemodynamic measurements of portal blood flow in our analysis. While we documented hepatopetal flow by ultrasound and venography, a quantitative assessment of portal hemodynamics was not performed.

In conclusion, in fasting patients with advanced cirrhosis, while ammonia concentrations differ significantly between the SMV and SV, there is no significant difference in ammonia concentration between the RPV and LPV. This finding challenges the hypothesis that differential ammonia shunting based on portal branch selection is a primary reason for variations in post-TIPS HE rates. Other factors, potentially related to overall shunt hemodynamics, preserved liver function, or individual patient susceptibility, may play a more dominant role and require further investigation.

## Methods

This prospective study was approved by the ethics committee of the Medical Faculty at the University of Bonn (application number 038/20), and all participants provided written informed consent. All methods were performed in accordance with the relevant guidelines and regulations.

### Patient population and study enrolment

Study enrolment is shown in Fig. 1. Participants were screened for TIPS creation between December 2021 and November 2023 (*n* = 220). From this pool, 50 consecutive participants were recruited who met the study inclusion criteria and provided adequate blood samples during the TIPS procedure.

As part of the evaluation of the TIPS procedure, neuropsychometric testing was performed using the psychometric hepatic encephalopathy score (PHES). The pre-TIPS imaging workup included cross-sectional imaging with at least two modalities: ultrasound and contrast-enhanced abdominal computed tomography or magnetic resonance imaging to exclude the presence of large spontaneous portosystemic shunts ≥ 8 mm in diameter. Hepatopetal blood flow in the main PV, SMV and SV was documented in all patients by ultrasound two days before the procedure and additionally by direct venogram during TIPS.

None of the patients received ammonia-lowering therapy (e.g., lactulose, rifaximin) prior to the TIPS procedure. Patients with advanced hepatocellular carcinoma or acute infection were excluded from the study.

### Clinical data collection

Prior to the TIPS procedure, the following clinical data were collected for each participant: demographic information (age, sex), etiology of liver cirrhosis, severity of liver disease (assessed by Model for End-Stage Liver Disease (MELD) score and Child-Pugh classification), the primary indication for TIPS, and results from pre-TIPS PHES testing.

### TIPS procedure and blood sampling protocol

All patients underwent a mandatory fasting period of at least six hours prior to the TIPS procedure. No other specific pre-procedural dietary restrictions were imposed.

The TIPS procedure was performed under fluoroscopic guidance. After obtaining transjugular access to the hepatic veins and subsequent ultrasound-guided puncture of the portal vein, a guidewire was advanced into the splanchnic venous system. A 4 F sizing catheter was then introduced over the wire into the portal vein. Standard portal and central venous pressure measurements were obtained, followed by a diagnostic portography.

Before balloon dilatation of the parenchymal tract and stent deployment, blood samples were meticulously collected. Using a 4 F end-hole diagnostic catheter, samples were sequentially aspirated from five pre-defined locations, guided by direct portography and fluoroscopic visualization to ensure optimal catheter tip placement:

1. **Right Portal Vein (RPV)**: From a segment within the right portal vein, clearly distal to the main portal vein bifurcation, ensuring the sample represented blood predominantly from the RPV territory.
2. **Left Portal Vein (LPV)**: From a segment within the left portal vein, clearly distal to the main portal vein bifurcation, ensuring the sample represented blood predominantly from the LPV territory.
3. **Main Portal Vein (PV)**: From the mid-portion of the main portal vein trunk, at a point judged to be approximately equidistant from the splenomesenteric confluence and the portal vein bifurcation.
4. **Superior Mesenteric Vein (SMV)**: From a segment within the superior mesenteric vein, clearly proximal to its confluence with the splenic vein, ensuring the sample was representative of SMV blood prior to mixing.
5. **Splenic Vein (SV)**: From a segment within the splenic vein, clearly proximal to its confluence with the superior mesenteric vein. If the inferior mesenteric vein (IMV) drained into the SV at a point distinctly proximal to the splenomesenteric confluence, the sample was obtained proximal to this IMV inflow to represent splenic venous blood before significant IMV contribution.

Patients with large spontaneous portosystemic shunts ≥ 8 mm were excluded from the study. If, during the procedure, relevant collateral veins were identified (particularly in patients undergoing TIPS for variceal bleeding), embolization was performed at the discretion of the interventional radiologist. In these cases, embolization was carried out prior to balloon dilatation and stent placement in order to reduce persistent portosystemic shunting and the risk of rebleeding. Blood sampling was consistently performed after embolization but before TIPS stent deployment.

To prevent sample dilution from saline flush or stagnant blood within the catheter, 5 mL of blood was aspirated and discarded from the catheter immediately before collecting each 2 mL diagnostic sample into an EDTA whole blood tube. All samples were kept at 4 °C and transported to the central laboratory for processing, with the pre-analytical time strictly limited to 15 min from collection to analysis.

### Blood analysis

Ammonia concentrations in the collected blood samples were determined using an enzymatic glutamate dehydrogenase method, measured with a UV/VIS photometer. The reagent utilized for this assay was NH3L2 from Roche Diagnostics.

### Statistical analysis and sample size calculation

Statistical analyses were conducted using SPSS version 26 (IBM, Armonk, New York, USA) and R (version 4.4.0; R Foundation for Statistical Computing, Vienna, Austria). Patient characteristics are presented as mean and standard deviation (SD) for normally distributed continuous variables, median and interquartile range (IQR) or range for non-normally distributed continuous variables, and frequencies (percentages) for categorical variables.

For the primary comparison between RPV and LPV ammonia concentrations, we used the Two One-Sided Tests (TOST) procedure to formally assess equivalence. This approach was chosen because our study hypothesis focused on whether ammonia levels in both portal branches can be considered practically the same. A conventional paired t-test would only detect statistically significant differences but cannot provide evidence for equivalence in the case of a non-significant result. In contrast, TOST allows testing whether the observed mean difference falls within a pre-specified equivalence margin, thereby directly addressing the hypothesis of “no relevant difference.” In the absence of prior data establishing a clinically significant difference in ammonia levels specifically between the RPV and LPV, and acknowledging this study’s exploratory nature in defining such a difference, an equivalence margin of ± 9% was prospectively determined. This margin was derived from a power calculation: a sample size of 50 paired measurements provides 80% power at a two-sided significance level of α = 0.05 to detect equivalence if the true mean difference lies within this ± 9% boundary. This threshold was chosen pragmatically to define a difference small enough to be considered practically negligible for the purposes of this initial investigation, recognizing that a definitive minimal clinically important difference for portal branch ammonia variation is not established in either animal or human studies.

Paired Student’s t-tests were used to assess differences in ammonia concentrations between the main PV, SMV, and SV. The formal test for equivalence between RPV and LPV ammonia levels utilized the TOST procedure with the aforementioned ± 9% equivalence boundary. The percentage difference for each RPV and LPV pair was calculated as [(RPV value - LPV value) / mean (RPV value, LPV value)] * 100. Bland-Altman analysis was performed to visually assess agreement and detect any systematic differences between RPV and LPV ammonia measurements. Pearson’s correlation coefficient was calculated to assess the linear relationship between RPV and LPV ammonia levels. For all inferential statistical tests, except the TOST procedure, a p-value < 0.05 was considered statistically significant. For the TOST procedure, a p-value < 0.05 indicates that the null hypothesis of non-equivalence can be rejected in favor of equivalence.

## Data Availability

The datasets generated and analysed during the current study are not publicly available due to them containing information that could compromise patient privacy. However, data are available from the corresponding author on reasonable request.
